# HIVprotI: an integrated web based platform for prediction and design of HIV proteins inhibitors

**DOI:** 10.1186/s13321-018-0266-y

**Published:** 2018-03-09

**Authors:** Abid Qureshi, Akanksha Rajput, Gazaldeep Kaur, Manoj Kumar

**Affiliations:** Bioinformatics Centre, Institute of Microbial Technology, Council of Scientific and Industrial Research, Sector 39A, Chandigarh, 160036 India

**Keywords:** HIV, Reverse transcriptase, Protease, Integrase, Inhibitors, QSAR, Algorithm, Web server

## Abstract

A number of anti-retroviral drugs are being used for treating Human Immunodeficiency Virus (HIV) infection. Due to emergence of drug resistant strains, there is a constant quest to discover more effective anti-HIV compounds. In this endeavor, computational tools have proven useful in accelerating drug discovery. Although methods were published to design a class of compounds against a specific HIV protein, but an integrated web server for the same is lacking. Therefore, we have developed support vector machine based regression models using experimentally validated data from ChEMBL repository. Quantitative structure activity relationship based features were selected for predicting inhibition activity of a compound against HIV proteins namely protease (PR), reverse transcriptase (RT) and integrase (IN). The models presented a maximum Pearson correlation coefficient of 0.78, 0.76, 0.74 and 0.76, 0.68, 0.72 during tenfold cross-validation on IC_50_ and percent inhibition datasets of PR, RT, IN respectively. These models performed equally well on the independent datasets. Chemical space mapping, applicability domain analyses and other statistical tests further support robustness of the predictive models. Currently, we have identified a number of chemical descriptors that are imperative in predicting the compound inhibition potential. HIVprotI platform (http://bioinfo.imtech.res.in/manojk/hivproti) would be useful in virtual screening of inhibitors as well as designing of new molecules against the important HIV proteins for therapeutics development.

**Electronic supplementary material:**

The online version of this article (10.1186/s13321-018-0266-y) contains supplementary material, which is available to authorized users.

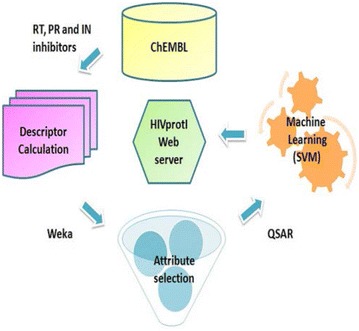

## Background

Human Immunodeficiency Virus (HIV) is one of the reasons for human death and suffering worldwide. It causes Acquired Immunodeficiency Syndrome (AIDS) in which gradual breakdown of the immune system allows critical opportunistic diseases to flourish [1]. As per the UNAIDS report, around 78 million people have become infected with HIV and 35 million people have died of AIDS-related illnesses since the start of the epidemic. In 2015 alone there were about 36.9 million people living with HIV of which 1.1 million died (http://www.unaids.org/en/resources/campaigns/HowAIDSchangedeverything/factsheet). Due to the high genetic variability and mutation rate of HIV, vaccines are not available to curb the HIV infection [2].

Researchers have put a considerable focus on HIV therapy and a lot of compounds have been tested against this pathogen [3, 4]. However, a few antiretroviral drugs have been able to slow the disease progression. These drugs blocked the function of proteins implicated in certain stages of the HIV life-cycle [5]. Different HIV enzymes are needed for the development of the retrovirus including reverse transcriptase (RT), protease (PR) and integrase (IN) [6]. RT creates complementary DNA from an RNA template which can integrate into the host genome and its inhibitors are widely used as antiretroviral drugs [7]. For example, the first anti-HIV drug zidovudine or azidothymidine (a nucleoside analog) was approved by the Food and Drug Administration (FDA) in 1987. It inhibits HIV reverse transcriptase, hence thwarting viral replication [8]. PR slices the newly synthesized polyproteins at the relevant positions to form the mature protein apparatus and is a major drug-target for treatment of HIV [9]. In 1995, saquinavir (invirase) became the first approved protease inhibitor. It blocks the enzyme’s active site, thus restricting the processing of HIV poly-proteins [10, 11]. The IN enzyme enables the virus to integrate its genetic material into the DNA of the host cell for a long-term infection. Compounds that inhibit the IN enzyme have demonstrated potent anti-HIV activity [12]. For example, raltegravir (isentress), the first integrase inhibitor was approved by FDA in 2007 [13]. Presently about 30 antiretroviral drugs are prescribed for the clinical treatment of AIDS [14]. An improved knowledge of the structure and function viral proteins has led antiviral drug developers to design better antivirals to treat HIV infections [15].

To conserve capital and time for finding novel drugs, scientists have extensively used different computational approaches to scan virtual compound libraries prior to the wet lab experiments [16]. The preferred targeted region should be off-target free and conserved across many strains of a virus for broad activity. Once the target is chosen, candidate antivirals can be selected by predicting the potential inhibitor using bioinformatics approaches [17, 18]. Amongst the diverse methods, quantitative structure activity relationship (QSAR) is being regularly used [19–22]. In QSAR, associations involving chemical descriptors and activity are employed to envisage the properties of other compounds [23]. The chemical descriptors present the structural information of a compound as numerical values [24]. Virtual screening employing QSAR is a valuable bioinformatics approach which helps to identify and devise of new antiviral drugs [25].

Several attempts have been made for predicting specific types of compounds against different HIV proteins (discussed later). Nevertheless, till date there no web server/software, which can regressively estimate the IC50/percentage inhibition activity of diverse types of, compounds against different HIV proteins. To accommodate this requirement, we created HIVprotI, a web based algorithm for prediction and design of protein specific anti-HIV compounds. In this approach, we employed experimentally validated inhibitors against RT, PR, IN (with IC_50_/percentage inhibition) from ChEMBL [26]. We calculated molecular descriptors and performed feature selection to pick the best performing descriptors, which were employed to build support vector machine (SVM) based QSAR models for the prediction of inhibitors against HIV proteins. We further incorporated the models in the HIVprotI web server, which will be useful for virtual screening and scheming novel inhibitors directed against HIV.

## Methods

### Datasets

In the present study, we have employed diverse datasets of inhibitors with experimentally validated IC_50_/percent inhibition activity against PR, RT and IN. The data was collected from ChEMBL resource (https://www.ebi.ac.uk/chembl/) by target browser (taxonomy tree) as well as target search using keywords like ‘Human Immunodeficiency Virus’, ‘HIV’, ‘protease’, ‘reverse transcriptase’ and ‘integrase’ etc. Initially among the inhibitors, majority of data belonged to RT, PR and IN with 3882, 3180, 2732 (IC_50_) and 1000, 740, 406 (percent inhibition) compounds respectively. After filtering entries with required information and eliminating redundant entries, we were left with 2126, 1895, 1240 (IC_50_) and 563, 518, 186 (percent inhibition) molecules correspondingly for the above mentioned proteins (hence 06 datasets) (Tables 1, 2). We have three times randomly picked ~ 10% of data as independent/validation dataset from each of six datasets. In each case this ~ 10% of the compounds were used for validation of the QSAR predictive models developed using the remaining 90% data during the training/testing [27]. This process is iterated three times for each of the six datasets and performances were comparable as detailed in the Additional file 1: Table S1. These datasets were employed for descriptor calculation and development of the models. The datasets can be accessed from the URLs: http://bioinfo.imtech.res.in/manojk/hivproti/ic50_datasets.php and http://bioinfo.imtech.res.in/manojk/hivproti/datasets.php.

**Table 1:** HIV protein inhibitor datasets used in the development of IC_50_ based QSAR models

Serial number	HIV protein	Overall data	Data filter		
			IC_50_	IC_50_ with reference	IC_50_ with reference and non-redundant
1	Protease	3180	2523	1963	1895
2	Reverse transcriptase	3882	2318	2222	2126
3	Integrase	2732	1296	1255	1240

Columns include HIV proteins overall data and filtered data (with quantitative inhibition value in terms of IC_50_) extracted from ChEMBL. Later incorporates redundant and non-redundant inhibitors (IC_50_) with a reference from a verifiable source

**Table 2:** HIV protein Inhibitor datasets used in the development of percent inhibition based QSAR models

Serial number	HIV protein	Overall data	Data filter		
			% inhibition	% inhibition with reference	% inhibition with reference and non-redundant
1	Protease	740	601	569	518
2	Reverse transcriptase	1000	943	921	563
3	Integrase	406	378	376	186

Columns include HIV proteins overall data and filtered data (with quantitative inhibition value in terms of %) extracted from ChEMBL. Later incorporates redundant and non-redundant inhibitors with a reference from a verifiable source

### Descriptor calculation

To develop protein specific prediction models, we calculated around 18,000 molecular descriptors which include geometric, electrostatic, structural, constitutional, path and graph fingerprints etc. utilizing the open source PaDEL software [28].

### Feature selection

To increase the speed of computation and eliminate unrelated features we chose the most necessary molecular descriptors employing the filter ‘RemoveUseless’ and attribute evaluator ‘ClassifierSubsetEval’ with ‘BestFirst’ as the search method in Waikato Environment for Knowledge Analysis (Weka) suite [29]. ClassifierSubsetEval estimates feature subsets on training/testing data utilizing a classifier to evaluate the worth of different feature sets.

### Machine learning

**Fig. 1:**
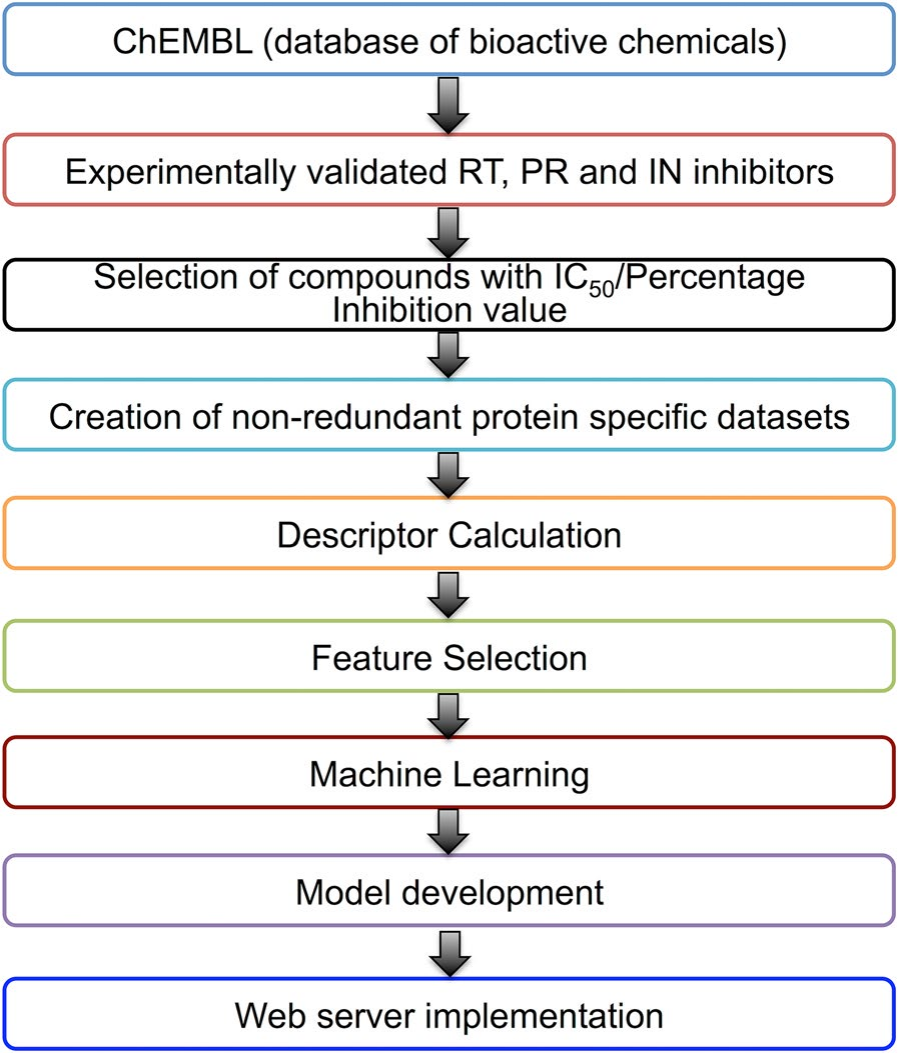
HIVprotI algorithm development

We created protein specific QSAR models for each of the 3-inhibitor classes (RT, PR and IN) employing SMOreg machine learning algorithm in Weka package. SMOreg executes the SVM in regression approach [29]. Chosen chemical descriptors and fingerprints were utilized for building the prediction models. The models were assessed by means of tenfold cross validation as well as independent validation [30]. The overall methodology for model development is depicted in Fig. 1. However, we used Tropsha’s validation test to statistically validate the prediction ability of developed model [31, 32].

### Evaluation

To calculate the performance of the QSAR models, we used various statistical measures such as Pearson’s correlation coefficient (PCC), Coefficient of Determination, Mean absolute error and Root-mean-square error described as follows.

*Pearson’s correlation coefficient (R)* calculates the correlation between two variables.

1 $$ {\text{R}} = \frac{{n\mathop \sum \nolimits_{n = 1}^{n} E_{i}^{act} E_{i}^{pred} - \mathop \sum \nolimits_{n = 1}^{n} E_{i}^{act} \mathop \sum \nolimits_{n = 1}^{n} E_{i}^{pred} }}{{\sqrt {n \mathop \sum \nolimits_{n = 1}^{n} \left( {E_{i}^{act} } \right)^{2} - \left( {\mathop \sum \nolimits_{n = 1}^{n} E_{i}^{act} } \right)^{2  } }   \sqrt {n \mathop \sum \nolimits_{n = 1}^{n} \left( {E_{i}^{pred} } \right)^{2} - \left( {\mathop \sum \nolimits_{n = 1}^{n} E_{i}^{pred} } \right)^{2} } }} $$

Here n is the size of test set while Ei^pred^ and Ei^act^ are the predicted and actual efficacies correspondingly.

A PCC value of 1 implies full positive correlation, 0 implies no correlation while − 1 implies full negative correlation.

*Coefficient of Determination (R*^*2*^*)* signifies how well a data fits the statistical model. An R^2^ value of 1 states that the model totally fits the data. On the other hand, a value of 0 implies that the model does not fit the data in any way.

*Mean absolute error (MAE)* calculates the closeness of predictions to the actual results.

2 $$ MAE = 1/n\mathop \sum \limits_{n = 1}^{n} |E_{i}^{pred} - E_{i}^{act} | $$

Here Ei^pred^ is the predicted value, Ei^act^ the true and |Ei^pred^–Ei^act^| the absolute error.

*Root-mean-square error (RMSE)* calculates the mean magnitude of the error.

3 $$ RMSE = \sqrt {1/n\mathop \sum \limits_{n = 1}^{n} (E_{i}^{pred} - E_{i}^{act} )^{2  } } $$

MAEs and RMSEs are negatively-oriented values, that is, lower values are superior.

### Chemical space mapping

Chemical space mapping of the datasets were done employing Ches-Mapper, a Java based application [33]. It involves creation of 3-dimensional structure (mmff94 force field), feature extraction (Chemical Development Kit (CDK) and hashed finger prints), clustering (k-means cascade method), embedding in 3-D space (or Dimensionality reduction) and alignment of compounds using maximum common subgraph (MCS). IC_50_ and percentage inhibition datasets of RT, PR and IN were individually mapped in the chemical space to understand the relationship between structure, physicochemical properties and biological aspects. All the clusters were provided as superimposed images with information of the number of sequences, 3-D embedding quality, and respective MCS.

### Applicability domain

Applicability domain (AD) of a QSAR model helps to measure its certainty in prediction [34]. We used Model Disturbance Index (MDI) v/s Prediction error (PE) method to calculate AD of all the prediction models [35]. It is calculated through Java-based Applicability Domain-Model Disturbance Index (AD-MDI) software (http://nanobridges.eu/software/). Validation datasets of RT, PR and IN for both IC_50_ and percentage inhibition were used to check their reliability on the respective training data sets. AD of the models was provided in form of scatter plots between MDI and PE.

## Results

### Performance of QSAR models

To facilitate the identification of the most efficient descriptors of anti HIV drugs against the three proteins, we calculated the correlation between chemical descriptors of the anti-HIV compounds and their IC_50_/percent inhibition. We have used wide-ranging datasets from the ChEMBL bioactivity resource [26]. After feature selection, the relevant descriptors that remained were 45, 61, 55 (IC_50_) and 42 49, 23 (percent inhibition) for PR, RT and IN respectively.

During tenfold cross validation, we attained maximum correlation (PCC) of 0.78, 0.76, 0.74 (IC_50_) and 0.76, 0.68, 0.72 (percent inhibition) in case of PR, RT and IN respectively. Further we reached a maximum PCC of 0.73, 0.72, 0.70 (IC_50_) and 0.70, 0.63, 0.65 (percent inhibition) on independent validation datasets for PR, RT, and IN correspondingly (Tables 3, 4).

**Table 3:** Performance of QSAR based predictive models developed on each of the HIV protein inhibitor (IC_50_) datasets during tenfold training/testing and on independent validation

Serial number	HIV protein	Inhibitor compounds			Number of selected descriptors	Pearson’s correlation coefficient (PCC)		*p* value
		Total	Training/testing	Independent validation		Training/testing (10×)	Independent validation	
1	Protease	1895	1706	189	45	0.78	0.73	1.00e−9
2	Reverse transcriptase	2126	1914	212	61	0.76	0.72	1.00e−7
3	Integrase	1240	1116	124	55	0.74	0.70	1.00e−6

**Table 4:** Performance of QSAR based predictive models developed on each of the HIV protein inhibitor (%) datasets during tenfold training/testing and on independent validation

Serial number	HIV protein	Inhibitor compounds			Number of selected descriptors	Pearson’s correlation coefficient (PCC)		*p* value
		Total	Training/testing	Independent validation		Training/testing (10×)	Independent validation	
1	Protease	518	466	52	42	0.76	0.70	1.00e−8
2	Reverse transcriptase	563	507	56	49	0.68	0.63	1.00e−7
3	Integrase	186	168	18	23	0.72	0.65	1.00e−3

**Fig. 2:**
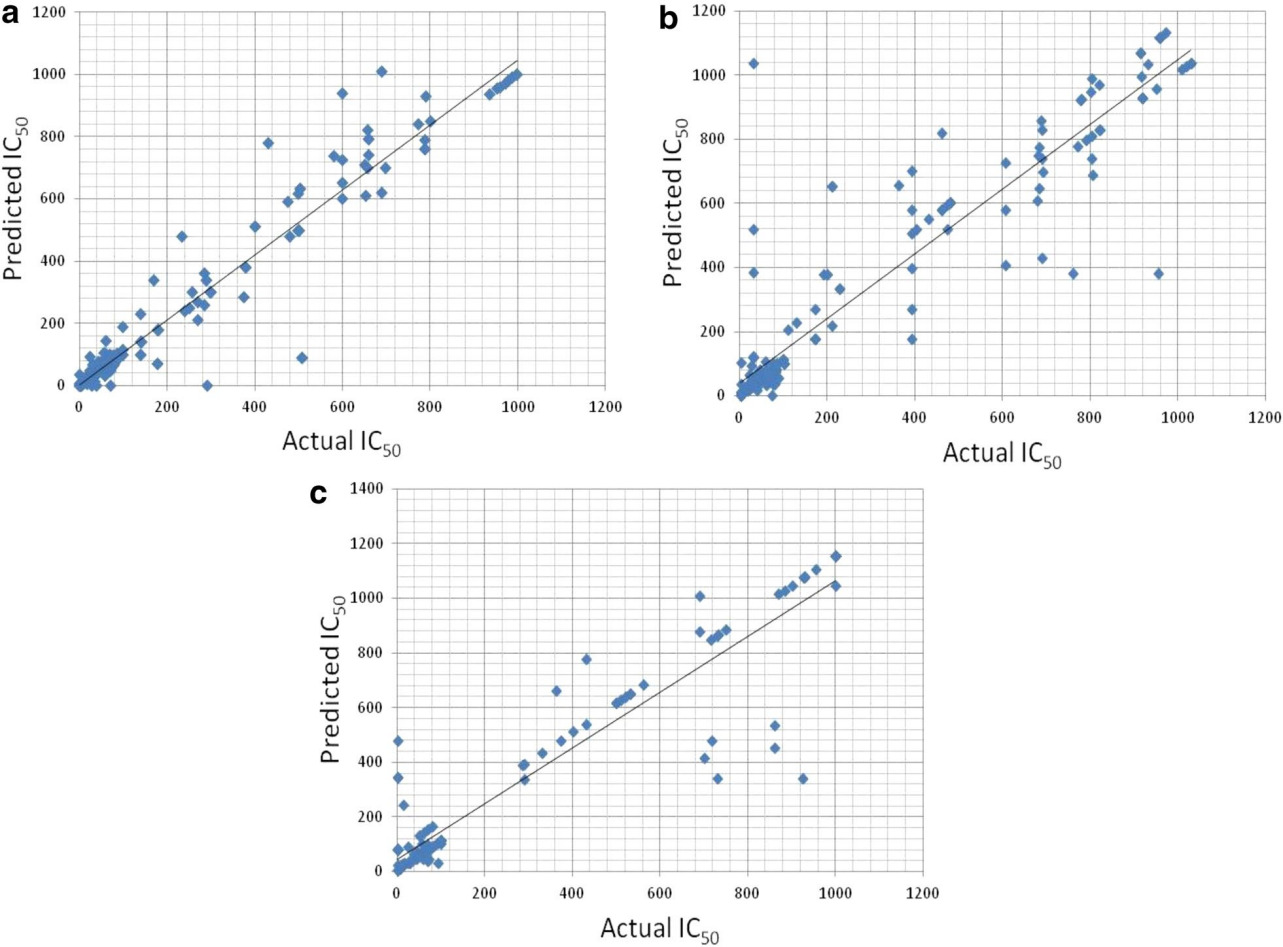
Scatter plot of predicted and actual IC_50_ (μM) on independent validation datasets of **a** reverse transcriptase (RT), **b** protease (PR) and **c** integrase (IN)

**Fig. 3:**
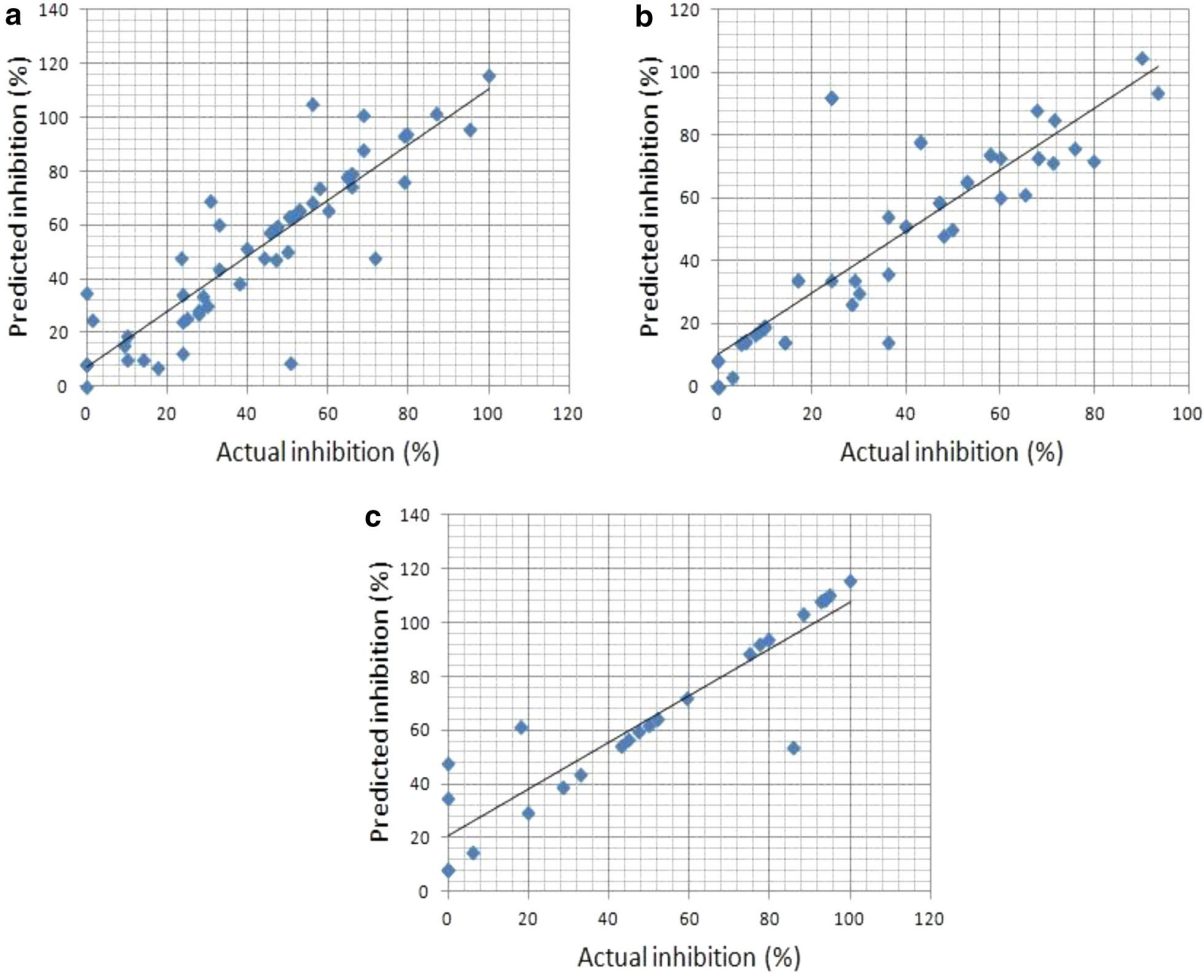
Scatter plot of predicted and actual percentage inhibition on independent validation datasets of **a** reverse transcriptase (RT), **b** protease (PR) and **c** integrase (IN)

Furthermore, all the models are statistically significant with *p* value < 0.001. Other statistical parameters used in the creation of prediction models are listed in Additional file 1: Tables S2 and S3. Scatter plots of predicted and actual activities are depicted in Figs. 2 and 3.

A compilation of chosen molecular descriptors such as atom type electrotopological state, partial charge, extended topochemical atom and several path/graph fingerprints were helpful in developing the algorithm (Additional file 1: Tables S4 and S5). The atom type electrotopological state holds information of electronic state of the bonded atom in a compound and its topological nature in the milieu of the entire molecular structure [36]. Likewise, the extended topochemical atom indices provide details of the electronic environment of the atoms, bonds, functional groups and branching [37] The details of other molecular descriptors have been discussed by Yap [28].

### Web server

The prediction models have been incorporated into an open source and simple to web application, ‘HIVprotI’. Here one can predict the inhibition activity of query compounds against the different HIV proteins in terms of IC_50_/percent inhibition. The web server components include:

#### Input

**Fig. 4:**
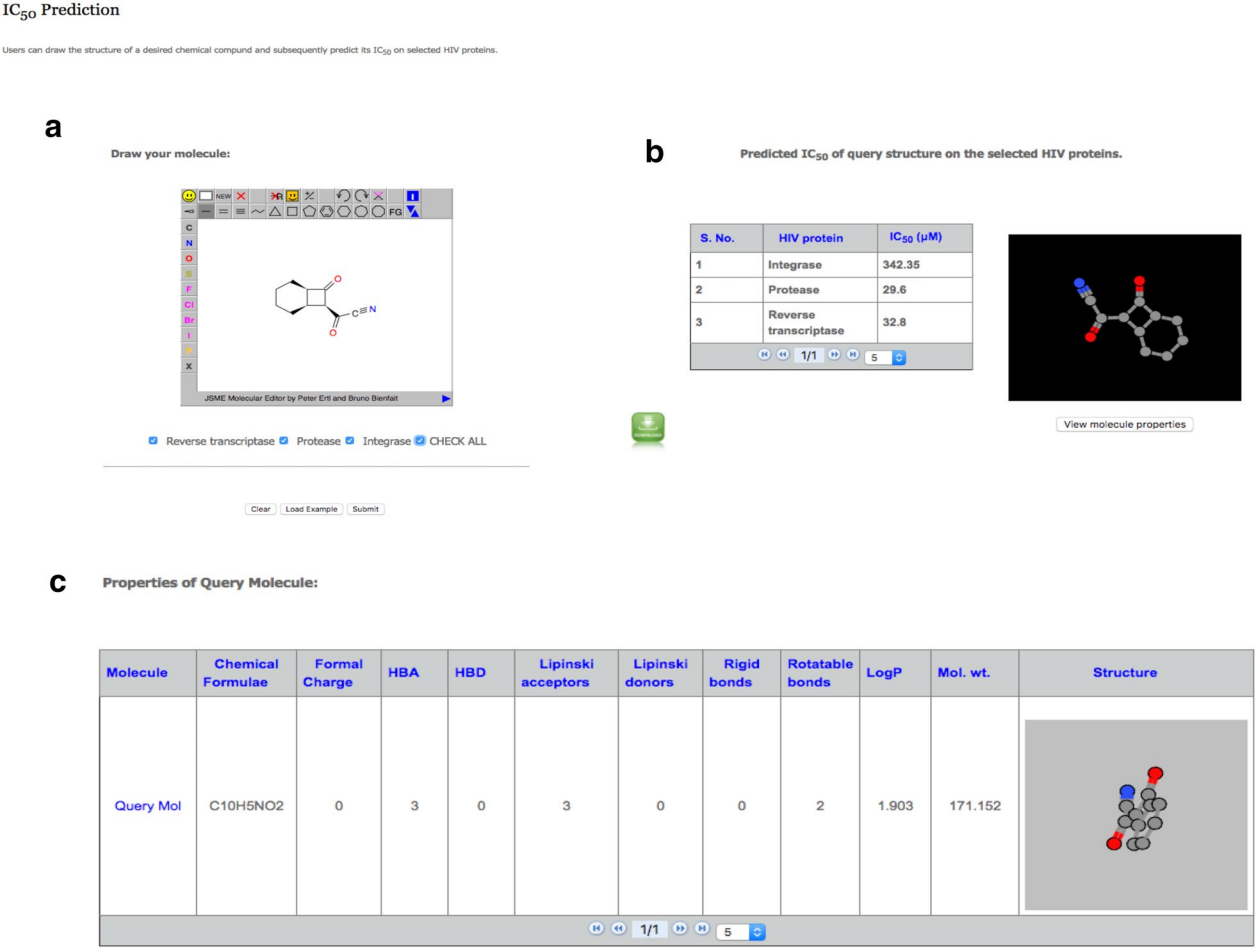
HIVprotI submission form (**a**) and result output (**b**, **c**)

Using this module one can submit/sketch the query molecule [38, 39]. Users can select the proteins on which they desire to virtually screen the query compound. Following the submission of an input molecule by the user, its structure is optimized using Obminimize programme (https://openbabel.org/wiki/Obminimize) to optimize the geometry and minimize the energy for a molecule before descriptor calculation and prediction [40]. On submission, it predicts IC_50_/percent inhibition activity against the HIV proteins. Users can also analyze the various properties of the query compound (Fig. 4).

#### Batch mode submission

Users can also submit multiple molecules simultaneously to check their inhibition efficiency against the desired HIV proteins. Clickable example molecules are given on the web server to help the users for easily getting started. This module will facilitate the researchers to virtually screen large number of compounds and select the ones with desired efficacy value. In addition, this component also enables the users to choose drug-like compounds by calculating the requisite properties. The batch mode can be accessed through the url: http://bioinfo.imtech.res.in/manojk/hivproti/batch.php.

#### Design analogs

Using this module, one can create analogs of their query structures based on user-defined components to evaluate the efficacy of the modified compounds on the selected HIV proteins. The structures are generated using SmiLib -a Java-based tool for rapid combinatorial library enumeration [41].

#### Output

The result output displays predicted IC_50_/percent inhibition activity against the chosen HIV proteins. In addition one can view the various chemical attributes of the query compound like structure, Hydrogen/Lipinski bond donors/acceptors, rotatable/rigid bonds, logP value etc. to recognize drug-like compounds (Fig. 4).

#### Search

HIVprotI also gives the users a search tool to find the data used in our study. In this component, the compounds targeting the PR, RT and IN proteins along with their structures are available in a database. The inhibitor entries can be easily searched and filtered from the web-application.

#### Implementation

HIVprotI has been created utilizing the open source Linux-Apache-MySQL-PHP (LAMP) server. The front-end of the web server was built using Hypertext Markup Language (HTML), Cascading Style Sheet (CSS), PHP: Hypertext Preprocessor (PHP) and JavaScript. The back-end of HIVprotI was created with Practical Extraction and Reporting Language (Perl), PHP and Structured Query Language (MySQL). The prediction software runs on Ubuntu 13 environment using Apache httpd server.

### Comparison with existing approaches

There are several QSAR methods exist for predicting various categories of HIV protein inhibitors, which are compared with HIVprotI algorithm as shown in Table 5. Nonetheless, these approaches are very specific and deal with a selected group of inhibitors such as quinolines [42], pyrimidones [43], processing inhibitors [44] etc. Owing to this rationale they envisage the compounds which are similar to the inhibitor class with a good correlation, but do not perform well on other structurally dissimilar inhibitors for the same HIV protein. Majority of such studies are based on a restricted quantity of inhibitors. Moreover, none of them till date have provided any web server/software to enable the researchers to screen AVCs or compare the output from different studies. To check the performance of existing methods we used their datasets and developed QSAR models using HIVprotI approach. Performance of such developed models during training/testing was similar to the reported one, but they did not perform well on our independent validation datasets. It can be due to the low number of compounds used in those studies and it is seen that the performance increased with the rise in the number and types of compounds (Table 6). Moreover, there are certain online resources like PASS Online (www.way2drug.com/passonline), SuperPred [45] and ChemProt server [46] for deciphering the biological potential and targets of different chemical compounds using information of existing drugs, environmental chemicals and natural products databases. In addition, AVCpred algorithm developed by our group helps in the prediction of generic antiviral compounds [47]. However, none of them is an HIV protein specific predictor. To address these limitations, HIVprotI has been developed employing more and varied inhibitors besides providing an open source integrated website for prediction and screening of protein specific anti-HIV compounds.

**Table 5:** Comparison of HIVprotI algorithm with existing QSAR based methods for predicting HIV proteins inhibitors

Serial number	Target	Predictive method and compounds type	Number of compounds	Correlation	Web server/software	Year	References
1	Protease	Non-peptide inhibitors	46	0.93–0.98	No	2010	[57]
2		Cycloalkylpyranone based compounds	170	0.6–0.83	No	2010	[58]
3		Ritonavir analogs	177	0.85	No	2012	[59]
4		Protease inhibitors	37	0.85–0.86	No	2015	[55]
5		Hydroxyethylamine derivatives	180	0.86	No	2015	[60]
6		Chemically diverse	1895	0.78	Yes	2017	HIVprotI
7	Reverse transcriptase	Amino-arylsulfonylbenzonitriles	68	0.86	No	2009	[61]
8		TIBO derivatives	70	0.83–0.88	No	2009	[62]
9		PETT derivatives	61	0.77–0.83	No	2009	[63]
10		HEPT derivatives	36	0.92	No	2011	[64]
11		Substituted benzoxazinones	33	0.8	No	2012	[65]
12		Non-nucleoside inhibitors	80	0.7–0.8	No	2014	[66]
13		Chemically diverse	2126	0.76	Yes	2017	HIVprotI
14	Integrase	Carboxylic acid derivatives	62	0.72–0.87	No	2010	[67]
15		N-methyl pyrimidones	51	0.84	No	2011	[43]
16		Quinoline ring derivatives	77	0.98	No	2012	[42]
17		Curcumine derivatives	39	0.91	No	2013	[68]
18		Chemically diverse	1240	0.74	Yes	2017	HIVprotI

**Table 6:** Comparison of HIVprotI approach based QSAR models developed on the datasets of the existing methods for predicting HIV proteins inhibitors (PR, Protease; RT, Reverse Transcriptase; IN, Integrase) and evaluation of both approaches on the independent validation datasets of HIVprotI

Serial number	Target	Compound type	Number of compounds	Correlation			Year	References
				Reported in the article	Observed by models developed using HIVprotI approach	On independent validation dataset of HIVprotI		
1	PR	Ritonavir analogs	177	0.85	0.81	0.31	2012	[59]
2		Cycloalkylpyran-one based compounds	70	0.60–0.83	0.73	0.40	2015	[58]
3	RT	Substituted benzoxazinones	33	0.80	0.74	0.23	2012	[65]
4		Non-nucleoside inhibitors	80	0.70–0.80	0.76	0.28	2014	[66]
5	IN	Quinoline ring derivatives	77	0.98	0.92	0.24	2012	[42]
6		Curcumine derivatives	39	0.91	0.88	0.16	2013	[68]

### Chemical clustering

**Fig. 5:**
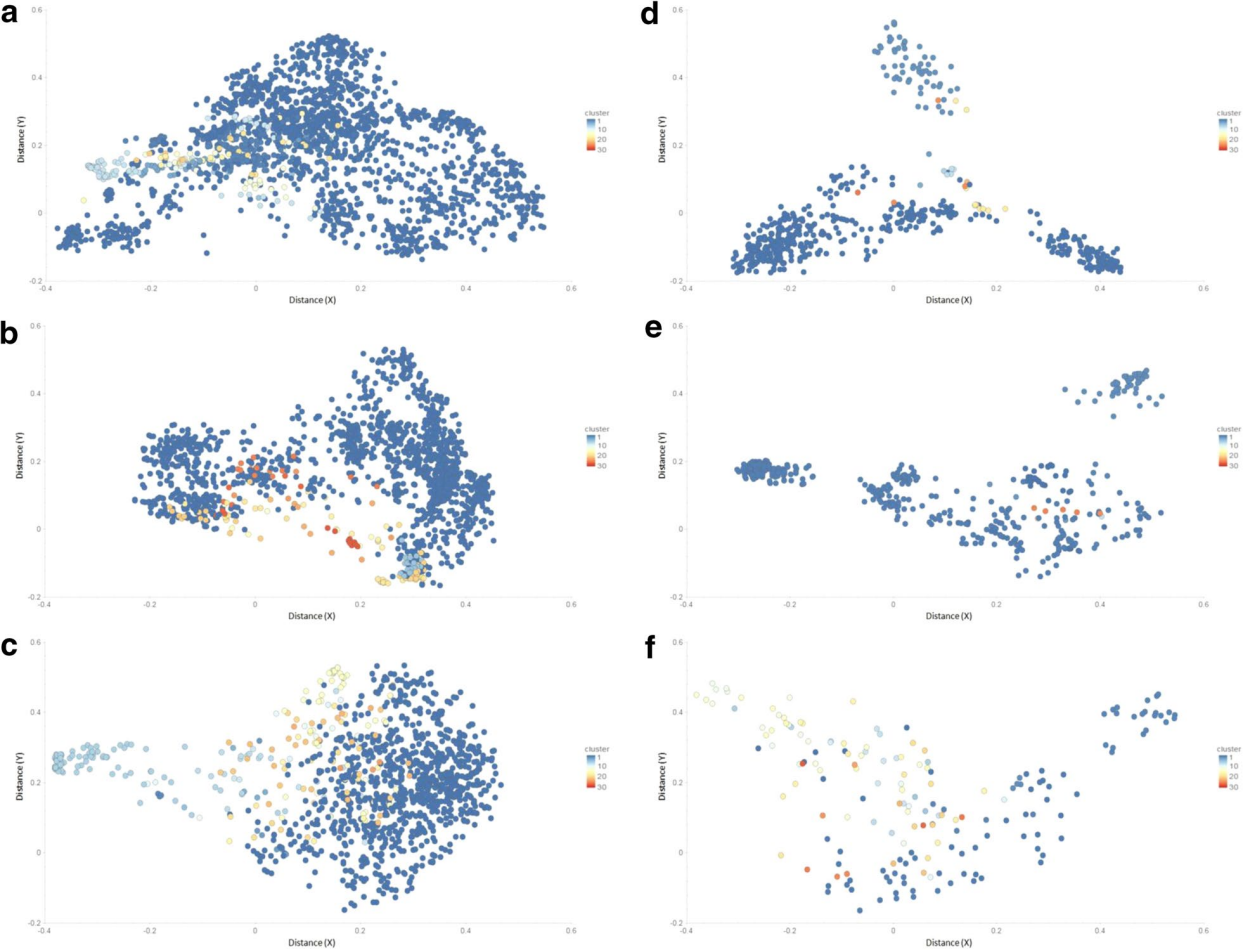
Chemical space analysis of IC_50_ **a**–**c** and percentage inhibition **d**–**f** based datasets for reverse transcriptase (RT), protease (PR) and integrase (IN) respectively

We used multidimensional scaling (MDS) to visualize the similitude of compounds in the different datasets. MDS calculates distance matrices by *all*-*against*-*all* evaluation of molecules from atom pair similarity values. The location or coordinates for each compound correspond to the distances graphically in a scatter plots. The plots were generated using Chemmine clustering workbench available at http://chemmine.ucr.edu/ [48]. Compounds that are similar are close to one another while dissimilar compounds are placed far apart. The clustering analysis revealed that the datasets employed in the development of IC_50_ based QSAR models are chemically more diverse compared to the percent inhibition ones (Fig. 5). Further the compounds targeting RT and IN were comparatively more dispersed in the chemical space than those directed against PR. Since the QSAR models developed in this study are more in number as well as type in comparison to other studies (Additional file 1: Figure S1), hence the algorithm will be better in predicting diverse types of HIV protein inhibitors (https://figshare.com/articles/Additional_file_2/5607103).

### Chemical space mapping

**Fig. 6:**
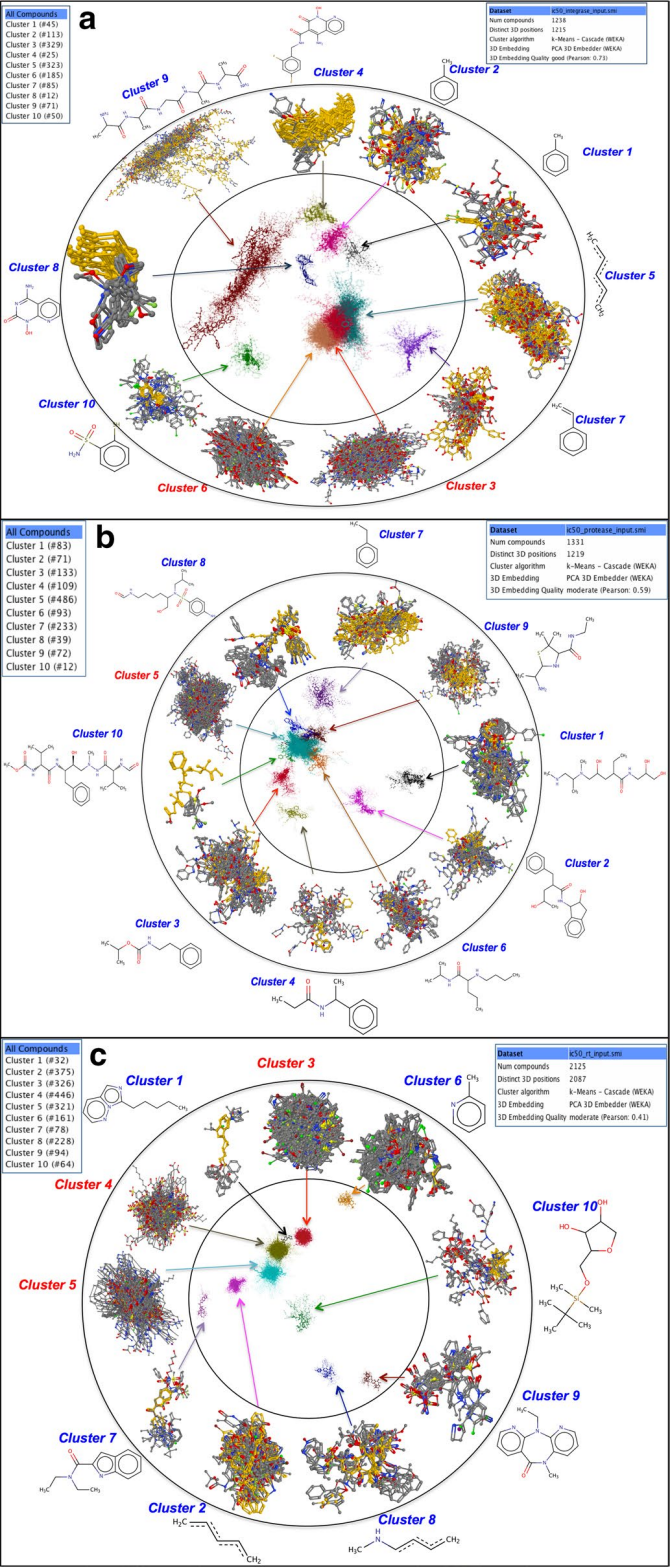
Chemical space mapping outline of **a** reverse transcriptase (RT), **b** protease (PR) and **c** integrase (IN) inhibitors (IC_50_) with internal circle showing clustering and 3-D embedding of compounds, middle circle with exact (zoomed) superimposed cluster and outermost circle with specific MCS of each cluster

Chemical space mapping uses 3-D embedding and clustering to display the similarity among compounds i.e. spatial proximity between two compounds based on their feature similarity. Chemical space mapping was done for each dataset of IC_50_ and percentage inhibition. Clustering was performed through k-means cascade algorithm. The datasets were grouped into subgroups, with similar feature values in each cluster. For example, in case of IN inhibitors (IC_50_) with 1238 compounds, there were 10 clusters with 45, 113, 329, 25, 323, 185, 85, 12, 71 and 50 molecules respectively. Figures integrating mapped 3-D superimposed clusters, outline of individual cluster, MCS of each cluster along with information of the number of molecules in each cluster is shown in Fig. 6 (IC_50_ datasets) and Additional file 1: Figure S2 (percentage inhibition datasets).

## Discussion

To hinder HIV proliferation, the anti-HIV compounds aim at important proteins of HIV that are involved in various steps of its life cycle such as replication, transcription, maturation, integration, etc. [2]. The drugs ought to be reasonably non-toxic to humans [49, 50]. The multiple stages of HIV life cycle can be inhibited employing compounds that can restrain viral enzymes like PR, RT, IN etc. that are requisite for HIV survival in the host cell. A growing list of these inhibitors are in clinical use and novel ones are also under trials [15].

Discovering innovative and enhanced drugs is a key objective in the management of HIV. Nevertheless, finding new inhibitors or compounds is a tedious procedure [51]. To accelerate the development of new inhibitors, computational methods employing QSAR approaches are widely employed to optimize the research budget prior to experimentation [24]. QSAR based algorithms have been extensively utilized in the selection of lead compounds and designing novel drugs [20].

In the present study, we created protein specific QSAR models to spot the probability of a given compound being an HIV protein inhibitor utilizing selected molecular descriptors of experimentally proven inhibitors against the HIV proteins. The open source PaDEL software was employed to compute numerous types of chemical descriptors followed by attribute selection approach to eliminate the irrelevant descriptors. We used machine learning to build the QSAR based models with good performance on various data sets of experimentally proven data from ChEMBL resource for specific HIV proteins. The developed models also displayed high performance while validated through independent data sets. Further, good predicting ability of the produced models was also observed by applying the statistical tests (Tropsha’s validation tests) for the continuous predictive models calculating Rext, k, k′ parameters as reported by (Additional file 1: Table S6) Golbraikh and Tropsha [31] and Vrontaki et al. [32]. Simultaneously, the robustness of the QSAR models was also examined using Y-randomization test by comparing their performance (Q^2^ and R^2^) to the models generated using randomized inhibition values. We noticed that models with high Q^2^ and R^2^ values developed with actual inhibition values were consistent whereas there was no model with high Q^2^ and R^2^ values developed with randomized inhibition values (Additional file 1: Table S7) [52, 53]. However, the algorithm can be enhanced with the availability of more high-throughput data on these enzymes in future.

Chemical clustering for each dataset were performed through multidimensional scaling (MDS) as well as k-means cascade algorithm. The clusters comprised of aromatic/ringed compounds like toluene, ethenyl benzene etc. Although each class of inhibitors has characteristic compounds but we found some common MCS between different data sets e.g. 1,2,4-pentatriene between IC_50_ datasets of IN and RT, benzene derivatives (e.g. ethenyl benzene) in IN and PR inhibitor datasets etc. This indicates that some inhibitors can have multiple targets. Hence, chemical space mapping would help to fetch the information about characteristic as well as common compounds among each class of inhibitors and further assist in finding broad-spectrum anti-HIV drugs.

**Fig. 7:**
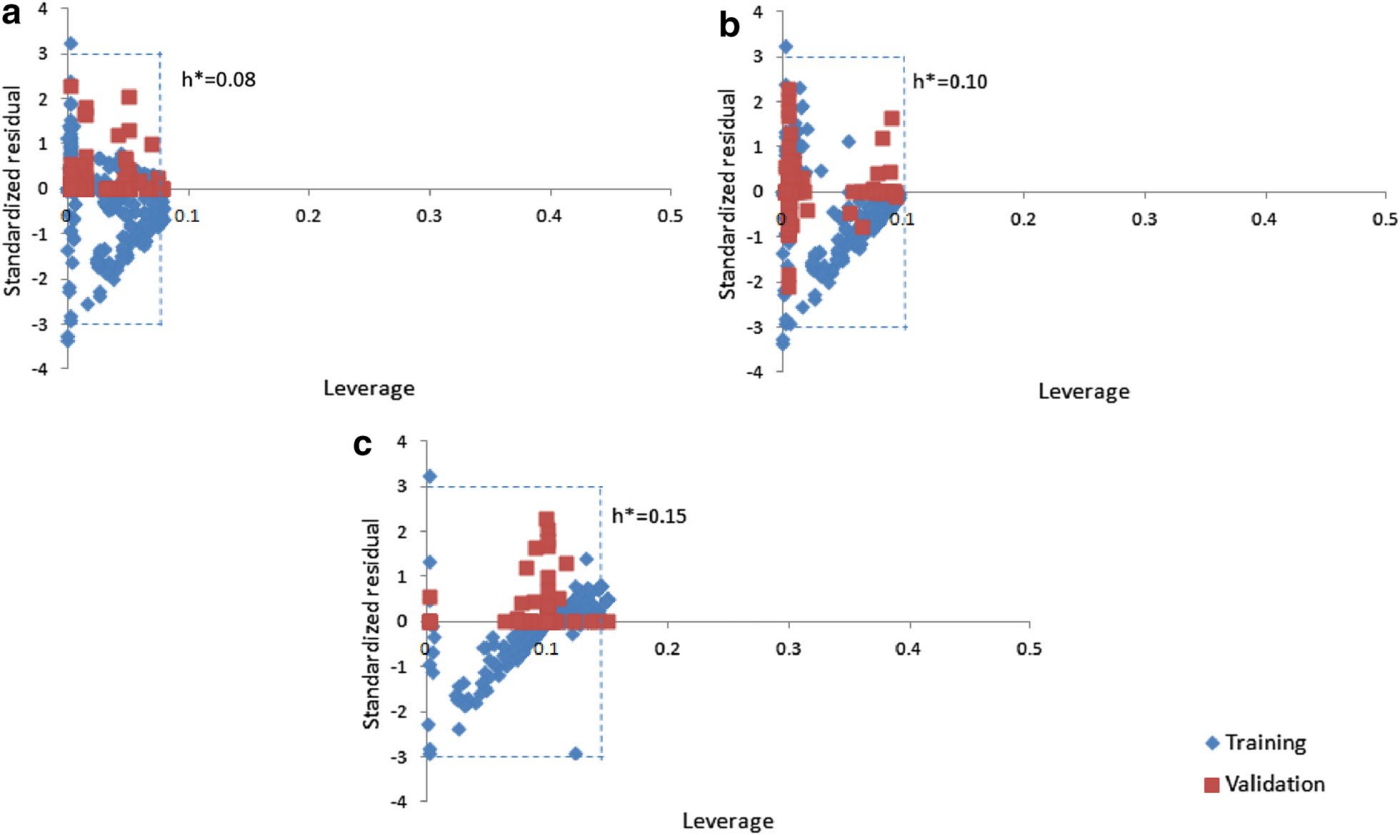
Applicability domain plots of the IC_50_ based QSAR models for **a** Reverse Transcriptase (RT), **b** Protease (PR) and **c** Integrase (IN)

**Fig. 8:**
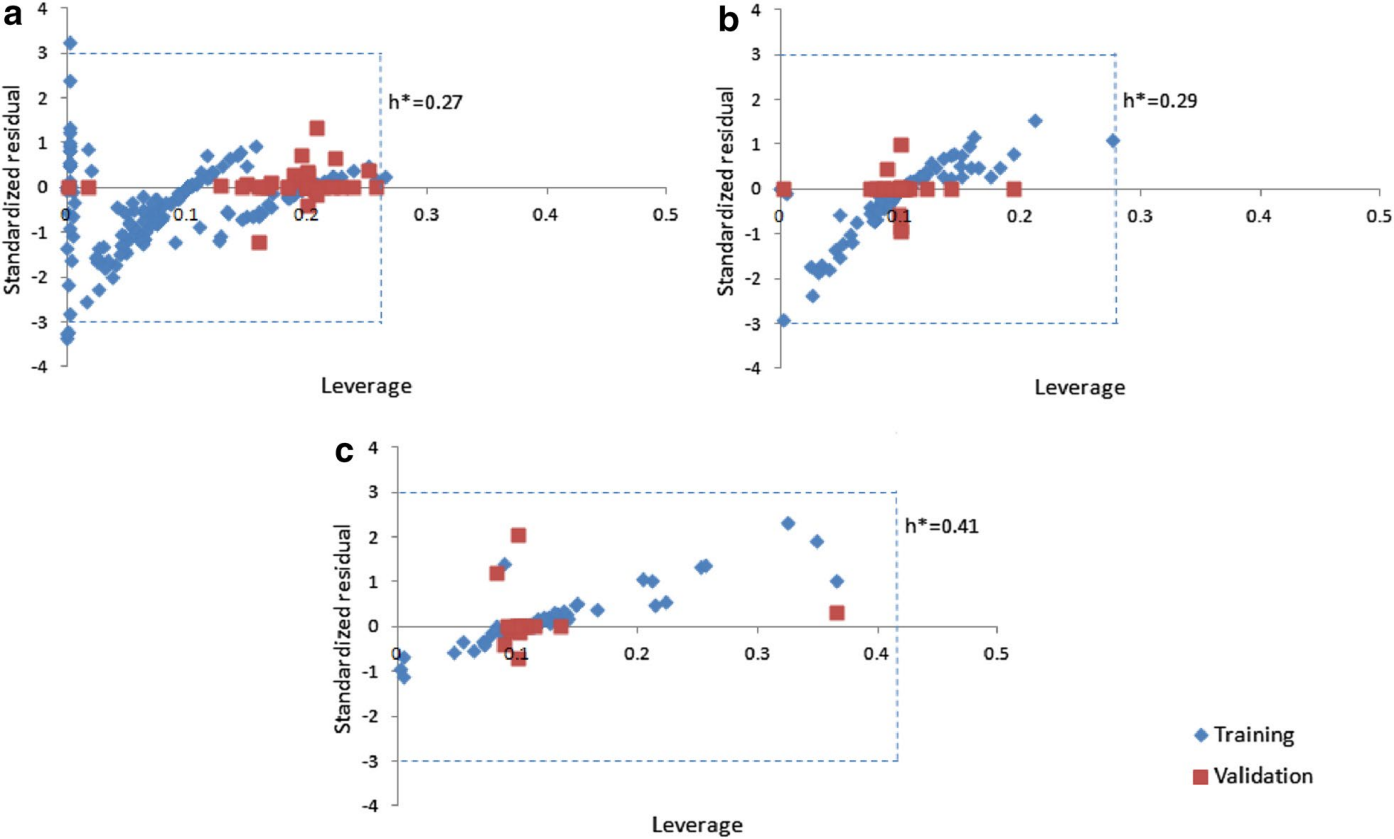
Applicability domain plots of the percentage inhibition based QSAR models for **a** reverse transcriptase (RT), **b** protease (PR) and **c** integrase (IN)

The applicability domain of the prediction models was verified by means of Williams plot (Figs. 7, 8) wherein standardized residuals are graphed against leverages [54]. If the standardized residual of a molecule is more than thrice the standard deviation (± 3σ), the molecule is considered to be an outlier. The caution value of leverage (h*) is computed as 3p/n, where p represents the number of selected descriptors plus one and n represents the number of compounds used in training [55, 56]. If the leverage of a compound is more than h*, it is labeled as an outlier. The plots reveal that the leverages of bulk of the molecules do not go beyond the caution value (h*) in the QSAR models and thus the applicability of the models is reasonable. AD was also evaluated on independent test data sets by model population approach i.e. by checking relationship between MDI and PE (Additional file 1: Figures S3 and S4). The scatter plots further confirm the reliability of the QSAR models with low outliers.

We also compared our prediction method with earlier algorithms and found that the latter are optimized to a specific class of inhibitors and do not perform well when tested with other types of inhibitors for the same target protein. Besides none of the earlier published methods provide a software or web server for the researchers. On the other hand, HIVprotI web server has useful services like sketching new compounds and estimate their inhibition activity against multiple HIV proteins. Users can also screen several molecules concurrently using batch mode module on the web server. In addition searchable databases of both experimental and predicted datasets are also provided. The HIVprotI algorithm will aid the researchers in envisaging new anti-HIV compounds and virtually analyze the outcome of alterations on current drugs.

## Conclusions

The HIVprotI is the first integrated web algorithm to predict anti-HIV compounds using experimentally verified data sets. Three QSAR prediction models for PR, RT and IN were developed to make all-inclusive predictions as well as screen compounds for their inhibition potential in a high throughput manner. The HIVprotI would be useful for scientists working in the field of anti-HIV therapeutics.

## Additional file

**Additional file 1.** Supporting information including **Table S1**. Performance of QSAR predictive models on three times randomly picked ~ 10% independent/validation data. These models were developed using remaining ~ 90% data during training/testing respectively for each of the six datasets; **Table S2**. Details of statistical parameters used for the development of IC_50_ based QSAR models; **Table S3**. Details of statistical parameters used for the development of percent inhibition based QSAR models; **Table S4**. Details of chemical descriptors used in the development of IC_50_ based QSAR models; **Table S5**. Details of chemical descriptors used in the development of percent inhibition based QSAR models; **Table S6**. Details of slopes k (predicted vs. observed inhibition) and k’ (observed vs. predicted inhibition) of the regression lines for the QSAR models; **Table S7**. Details of Y-randomization test performed on the QSAR models; **Figure S1**. Chemical space analysis of QSAR studies (Table 5) for Protease (PR) (a, b), Reverse Transcriptase (RT) (c, d) and Integrase (IN) (e, f) respectively; **Figure S2**. Chemical space mapping outline of (a) Integrase (IN), (b) Protease (PR) and (c) Reverse Transcriptase (RT) inhibitors (percentage inhibition) with internal circle showing clustering and 3-D embedding of compounds, middle circle with exact (zoomed) superimposed cluster and outermost circle with specific MCS of each cluster; **Figure S3**. Scatter plot depicting the applicability domain for IC_50_ datasets of (a) Integrase (IN), (b) Protease (PR) and (c) Reverse Transcriptase (RT); **Figure S4**. Scatter plot depicting the applicability domain for percentage inhibition datasets of (a) Integrase (IN), (b) Protease (PR) and (c) Reverse Transcriptase (RT).

**Additional file 2.** Source code of HIVProtI web server.

## Abbreviations

AIDS: Acquired Immunodeficiency Syndrome
FDA: Food and Drug Administration
HIV: Human Immunodeficiency Virus
IN: integrase
MAE: mean absolute error
MDS: multidimensional scaling
PR: protease
QSAR: quantitative structure-activity relationship
RMSE: root-mean-square error
RT: reverse transcriptase
SMILES: simplified molecular-input line-entry system
SVM: support vector machine
