# Supplementary material for: HIVprotI: an integrated web based platform for prediction and design of HIV proteins inhibitors
Source: J Cheminform. 2018 Mar 9;10:12. doi: 10.1186/s13321-018-0266-y (PMC5845081; doi:10.1186/s13321-018-0266-y)
Supplement: Supplementary file 1 — Additional file 1. Supporting information including Table S1. Performance of QSAR predictive models on three times randomly picked ~ 10% independent/validation data. These models were developed using remaining ~ 90% data during training/testing respectively for each of the six datasets; Table S2. Details of statistical parameters used for the development of IC50 based QSAR models; Table S3. Details of statistical parameters used for the development of percent inhibition based QSAR models; Table S4. Details of chemical descriptors used in the development of IC50 based QSAR models; Table S5. Details of chemical descriptors used in the development of percent inhibition based QSAR models; Table S6. Details of slopes k (predicted vs. observed inhibition) and k’ (observed vs. predicted inhibition) of the regression lines for the QSAR models; Table S7. Details of Y-randomization test performed on the QSAR models; Figure S1. Chemical space analysis of QSAR studies (Table 5) for Protease (PR) (a, b), Reverse Transcriptase (RT) (c, d) and Integrase (IN) (e, f) respectively; Figure S2. Chemical space mapping outline of (a) Integrase (IN), (b) Protease (PR) and (c) Reverse Transcriptase (RT) inhibitors (percentage inhibition) with internal circle showing clustering and 3-D embedding of compounds, middle circle with exact (zoomed) superimposed cluster and outermost circle with specific MCS of each cluster; Figure S3. Scatter plot depicting the applicability domain for IC50 datasets of (a) Integrase (IN), (b) Protease (PR) and (c) Reverse Transcriptase (RT); Figure S4. Scatter plot depicting the applicability domain for percentage inhibition datasets of (a) Integrase (IN), (b) Protease (PR) and (c) Reverse Transcriptase (RT). [file 13321_2018_266_MOESM1_ESM.pdf]

HIVprotI: An integrated web based platform for prediction and design of HIV proteins inhibitors

Abid Qureshi, Akanksha Rajput, Gazaldeep Kaur and Manoj Kumar*

Bioinformatics Centre, Institute of Microbial Technology, Council of Scientific and Industrial Research, Sector 39-A, Chandigarh-160036, India

*Correspondence to: Manoj Kumar (E-mail: manojk@imtech.res.in)

Additional files

Tables

Table S1. Performance of QSAR predictive models on three times randomly picked ~10% independent/validation data. These models were developed using remaining ~90% data during training/testing respectively for each of the six datasets.

Table S2. Details of statistical parameters used for the development of IC_{50} based QSAR models.

Table S3. Details of statistical parameters used for the development of percent inhibition based QSAR models.

Table S4. Details of chemical descriptors used in the development of IC_{50} based QSAR models.

Table S5. Details of chemical descriptors used in the development of percent inhibition based QSAR models.

Table S6. Details of slopes k (predicted versus observed inhibition) and k' (observed versus predicted inhibition) of the regression lines for the QSAR models

Table S7. Details of Y-randomization test performed on the QSAR models

Figures

Figure S1. Chemical space analysis of QSAR studies (Table 5) for Protease (PR) (a, b), Reverse Transcriptase (RT) (c, d) and Integrase (IN) (e, f) respectively.

Figure S2. Chemical space mapping outline of a) Integrase (IN), b) Protease (PR) and c) Reverse Transcriptase (RT) inhibitors (percentage inhibition) with internal circle showing clustering and 3-D embedding of compounds, middle circle with exact (zoomed) superimposed cluster and outermost circle with specific MCS of each cluster.

Figure S3. Scatter plot depicting the applicability domain for IC_{50} datasets of a) Integrase (IN), b) Protease (PR) and c) Reverse Transcriptase (RT).

Figure S4. Scatter plot depicting the applicability domain for percentage inhibition datasets of a) Integrase (IN), b) Protease (PR) and c) Reverse Transcriptase (RT).

Table S1. Performance of QSAR predictive models on three times randomly picked ~10% independent/validation data. These models were developed using remaining ~90% data during training/testing respectively for each of the six datasets.

Protein	Model No.	Pearson's correlation coefficient (R)		Data Type
		Training/ Testing	Validation	
1. Reverse transcriptase	**RT-IC_{50}-random1**	**0.76**	**0.72**	IC_{50}
	RT-IC_{50}-random2	0.70	0.71	
	RT-IC_{50}-random3	0.71	0.71	
2. Protease	**PR-IC_{50}-random1**	**0.78**	**0.73**	
	PR-IC_{50}-random2	0.72	0.71	
	PR-IC_{50}-random3	0.75	0.71	
3. Integrase	**IN-IC_{50}-random1**	**0.74**	**0.70**	
	IN-IC_{50}-random2	0.66	0.67	
	IN-IC_{50}-random3	0.64	0.69	
4. Reverse transcriptase	**RT-PI-random1**	**0.68**	**0.63**	Percent inhibition
	RT-PI-random2	0.64	0.62	
	RT-PI-random3	0.62	0.60	
5. Protease	**PR-PI-random1**	**0.76**	**0.70**	
	PR-PI-random2	0.71	0.69	
	PR-PI-random3	0.71	0.68	
6. Integrase	**IN-PI-random1**	**0.72**	**0.65**	
	IN-PI-random2	0.66	0.63	
	IN-PI-random3	0.65	0.64	

Table S2. Details of statistical parameters used for the development of IC_{50} based QSAR models.

S. No.	Protein	Training				Validation			
		**R**	**R^2**	**MAE**	**RMSE**	**R**	**R^2**	**MAE**	**RMSE**
1	Protease	0.78	0.61	10.21	12.31	0.73	0.53	12.52	15.24
2	Reverse transcriptase	0.76	0.58	10.43	14.21	0.72	0.52	14.05	16.10
3	Integrase	0.74	0.55	11.84	15.04	0.70	0.49	15.09	16.92

R :Pearson correlation coefficient. It is a measure of correlation between two variables. A value of 1 denotes total positive correlation, 0 is no correlation and −1 is total negative correlation. **R^2 :Coefficient of determination**. It indicates how well data fit a statistical model. An R^2 of 1 indicates that the model perfectly fits the data, while an R^2 of 0 means that the model does not fit the data at all. **MAE :Mean absolute error.** This measure indicates how close the predictions are to the eventual outcomes. MAEs are negatively-oriented scores, that is, lower values are better. **RMSE :Root-mean-square error** measures the average magnitude of the error. RMSEs are also negatively-oriented scores, that is, lower values are better.

Table S3. Details of statistical parameters used for the development of percent inhibition based QSAR models.

S. No.	Protein	Training				Validation			
		**R**	**R^2**	**MAE**	**RMSE**	**R**	**R^2**	**MAE**	**RMSE**
1	Protease	0.76	0.58	10.51	14.12	0.70	0.49	11.75	18.34
2	Reverse transcriptase	0.68	0.46	15.42	23.16	0.63	0.40	19.21	27.12
3	Integrase	0.72	0.52	12.82	16.41	0.65	0.42	17.49	25.19

R: Pearson correlation coefficient. It is a measure of correlation between two variables. A value of 1 denotes total positive correlation, 0 is no correlation and −1 is total negative correlation. **R^2**: Coefficient **of determination**. It indicates how well data fit a statistical model. An R^2 of 1 indicates that the model perfectly fits the data; while an R^2 of 0 means that the model does not fit the data at all. **MAE: Mean absolute error.** This measure indicates how close the predictions are to the eventual outcomes. MAEs are negatively-oriented scores, that is, lower values are better. **RMSE: Root-mean-square error** measures the average magnitude of the error. RMSEs are also negatively-oriented scores, that is, lower values are better.

Table S4. Details of chemical descriptors used in the development of IC_{50} based QSAR models.

Descriptor	Type	Details
BCUTc-1h	BCUT	nlow highest partial charge weighted BCUTS
EStateFP35	EState Fingerprinter	[OD1H0]=*
ExtFP129, ExtFP160, ExtFP184, ExtFP237, ExtFP258, ExtFP275, ExtFP279, ExtFP284, ExtFP365, ExtFP409, ExtFP425, ExtFP434, ExtFP436, ExtFP440, ExtFP457, ExtFP483, ExtFP489, ExtFP491, ExtFP5, ExtFP52, ExtFP534, ExtFP597, ExtFP632, ExtFP634, ExtFP651, ExtFP666, ExtFP687, ExtFP695, ExtFP747, ExtFP75, ExtFP786, ExtFP811, ExtFP818, ExtFP872, ExtFP873, ExtFP877, ExtFP9, ExtFP920, ExtFP949, ExtFP955, ExtFP957, ExtFP978	Extended Fingerprinter	Extends the Fingerprinter with additional bits describing ring features
FP100, FP1022, FP111, FP117, FP142, FP147, FP181, FP183, FP19, FP230, FP289, FP338, FP346, FP374, FP385, FP396, FP416, FP419, FP483, FP490, FP510, FP549, FP551, FP555, FP560, FP587, FP60, FP601, FP602, FP630, FP632, FP666, FP762, FP763, FP77, FP822, FP835, FP862, FP865, FP872, FP903	Fingerprinter	Path based fingerprints
GraphFP101, GraphFP1010, GraphFP154, GraphFP243, GraphFP262, GraphFP374, GraphFP396, GraphFP428, GraphFP432, GraphFP441, GraphFP443, GraphFP46, GraphFP541, GraphFP575, GraphFP589, GraphFP599, GraphFP612, GraphFP616, GraphFP62, GraphFP700, GraphFP911, GraphFP942, GraphFP964, GraphFP997	Graph Only Fingerprinter	Specialized version of the Fingerprinter which does not take bond orders into account
KRFP126	Klekota-Roth fingerprint	[!#1][CH]=[CH][!#1]
KRFP1430		[!#1]C(=O)c1[cH][cH][cH][cH]c1[OH]
KRFP1642		[!#1]c1[cH][cH]c([!#1])[cH][cH]1
KRFP3591		Cc1cccc(O)c1
KRFP3592		Cc1cccc2ccccc12
KRFP371		[!#1][CH2][CH2][CH2]C(=O)O[CH3]
KRFP3721		CCCCCCC=O
KRFP390		[!#1][CH2][CH2][NH]C(=O)[CH2][!#1]
KRFP3940		CNC=C
KRFP3971		COC=O

KRFP4045		FC(F)(F)c1cccc(NC=O)c1
KRFP4468		O=CC(=O)c1ccccc1
KRFP4612		OC(=O)C=Cc1ccccc1
KRFP4681		OC(=O)CS
KRFP585		[!#1][CH2]c1[cH][cH]c([CH3])[cH][cH]1
KRFP590		[!#1][CH2]c1[cH][cH]c(F)[cH][cH]1
KRFP74		[!#1][CH]([CH]([CH3])[CH3])C(=O)[OH]
KRFP992		[!#1][NH]c1[cH][cH][cH]c([!#1])[cH]1
KRFPC3224		CC
KRFPC3646		CCC(=O)CC
KRFPC3704		CCCc1ccccc1
KRFPC3710		CCCCc1ccccc1
KRFPC3730		CCCCCO
KRFPC4740		Oc1ccc2ccccc2c1
MACCSFP87	MACCS fingerprint	('[F,Cl,Br,I]!@*@*',0), # X!A$A
maxsSH	Atom type electrotopological state	Maximum atom-type E-State: -SH
nHBint10		Count of E-State descriptors of strength for potential Hydrogen Bonds of path length 10
SwHBd		Sum of E-States for weak Hydrogen Bond donors
SaaS		Sum of atom-type E-State: aSa
SdO		Sum of atom-type E-State: =O
SsF		Sum of atom-type E-State: -F
PubchemFP152	Pubchem fingerprint	>= 2 saturated or aromatic nitrogen-containing ring size 5
PubchemFP21		>= 8 O
PubchemFP38		>= 2 Cl
PubchemFP383		C(~S)(:C)
PubchemFP434		C(-C)(-H)(=C)
PubchemFP441		C(-C)(=C)
PubchemFP540		C-N-C-[#1]
PubchemFP644		C-C=N-N-C
PubchemFP703		O-C-C-C-C-C(N)-C
PubchemFP707		O=C-C-C-C-C(N)-C
PubchemFP742		Oc1cc(N)ccc1
MDEO-12	Molecular distance edge	Molecular distance edge between all primary and secondary oxygens
nHBAcc3	Hbond acceptor count	Number of hydrogen bond acceptors (any oxygen; any nitrogen where the formal charge of the nitrogen is non-positive (i.e. formal charge <= 0) except a non-

		aromatic nitrogen that is adjacent to an oxygen and aromatic ring, or an aromatic nitrogen with a hydrogen atom in a ring, or an aromatic nitrogen with 3 neighouring atoms in a ring, or a nitrogen with total bond order >=4, or a nitrogen in an amide bond; any fluorine)
SCH-5	Chi chain descriptor	Simple chain, order 5
SubFP10	Substructure Fingerprinter	[BrX1][CX4]
SubFP302		[!$(*#*)&!D1]-!@[!$(*#*)&!D1]
SubFPC246	Substructure fingerprint count	[PX4D4](=[!#6])([!#6])([!#6])[!#6]

Table S5. Details of chemical descriptors used in the development of percent inhibition based QSAR models.

Descriptor	Type	Details
BCUTc-1l	BCUT	nhigh lowest partial charge weighted BCUTS
ETA_Beta_s	Extended topochemical atom	A measure of electronegative atom count of the molecule
ETA_dEpsilon_B		A measure of contribution of unsaturation
ExtFP137, ExtFP144, ExtFP160, ExtFP163, ExtFP167, ExtFP194, ExtFP204, ExtFP262, ExtFP325, ExtFP409, ExtFP415, ExtFP460, ExtFP469, ExtFP484, ExtFP497, ExtFP535, ExtFP604, ExtFP619, ExtFP657, ExtFP677, ExtFP748, ExtFP89, ExtFP934, ExtFP992	Extended Fingerprinter	Extends the Fingerprinter with additional bits describing ring features
FP138, FP16, FP169, FP18, FP196, FP225, FP227, FP297, FP37, FP465, FP47, FP494, FP498, FP505, FP520, FP541, FP617, FP645, FP773, FP777, FP782, FP819, FP872, FP911, FP923, FP986, FP990	Fingerprinter	Path based fingerprints
GraphFP101, GraphFP199, GraphFP212, GraphFP251, GraphFP252, GraphFP271, GraphFP368, GraphFP387, GraphFP523, GraphFP660, GraphFP826, GraphFP87, GraphFP895, GraphFP944	Graph Only Fingerprinter	Specialized version of the Fingerprinter which does not take bond orders into account
KRFP3529	Klekota-Roth fingerprint	Cc1ccc(C)cc1
KRFP3590		Cc1cccc(NC=O)c1
KRFP3742		CCCNC=O
MACCSFP93	MACCS fingerprint	('[!#6;!#1]~[CH3]',0), # QCH3
maxHBint8	Atom type electrotopological state	Maximum E-State descriptors of strength for potential Hydrogen Bonds of path length 8
maxHother		Maximum atom-type H E-State: H on aaCH, dCH2 or dsCH
nssssC		Minimum atom-type E-State: >C<
SsCl		Sum of atom-type E-State: -Cl
SsssN		Sum of atom-type E-State:

		>N-
PubchemFP497	Pubchem fingerprint	O-C:C:N
PubchemFP638		O-C-C-C-N
PubchemFP639		O-C-C-C-O
SC-5	Chi cluster	Simple cluster, order 5
SubFP32	Substructure fingerprint	[NX3H1+0,NX4H2+;$([N]([c])[C]);!$([N]*~[#7,#8,#15,#16])]
VPC-6	Chi path cluster	Valence path cluster, order 6
WTPT-1	Weighted path	Molecular ID
WTPT-5		Sum of path lengths starting from nitrogens

Table S6. Details of slopes k (predicted versus observed inhibition) and k' (observed versus predicted inhibition) of the regression lines for the QSAR models

S. No.	Protein	k	k'	Data Type
1	Reverse transcriptase	0.75	0.73	IC_{50}
2	Protease	0.72	0.74	
3	Integrase	0.78	0.80	
4	Reverse transcriptase	0.67	0.68	Percent inhibition
5	Protease	0.67	0.69	
6	Integrase	0.60	0.62	

Table S7. Details of Y-randomization test performed on the QSAR models

Protein	Model No.	Q^2		R^2		Data Type
		Actual activity	Randomized activity	Actual activity	Randomized activity	
Reverse transcriptase	RT-IC_{50}-random1	0.58	0.04	0.52	0.01	IC_{50}
	RT-IC_{50}-random2	0.49	0.01	0.50	0.03	
	RT-IC_{50}-random3	0.50	0.01	0.51	0.01	
Protease	PR-IC_{50}-random1	0.61	0.02	0.53	0.05	
	PR-IC_{50}-random2	0.52	0.04	0.51	0.02	
	PR-IC_{50}-random3	0.56	0.03	0.50	0.03	
Integrase	IN-IC_{50}-random1	0.55	0.02	0.49	0.04	
	IN-IC_{50}-random2	0.43	0.01	0.45	0.01	
	IN-IC_{50}-random3	0.41	0.05	0.48	0.05	
Reverse transcriptase	RT-PI-random1	0.46	0.02	0.40	0.02	Percent inhibition
	RT-PI-random2	0.41	0.03	0.38	0.04	
	RT-PI-random3	0.39	0.04	0.36	0.01	
Protease	PR-PI-random1	0.58	0.01	0.49	0.01	
	PR-PI-random2	0.51	0.05	0.47	0.02	
	PR-PI-random3	0.50	0.02	0.46	0.04	
Integrase	IN-PI-random1	0.52	0.03	0.42	0.03	
	IN-PI-random2	0.44	0.01	0.40	0.02	
	IN-PI-random3	0.42	0.03	0.41	0.03	

Figure S1. Chemical space analysis of QSAR studies (Table 5) for Protease (PR) (a, b), Reverse Transcriptase (RT) (c, d) and Integrase (IN) (e, f) respectively.

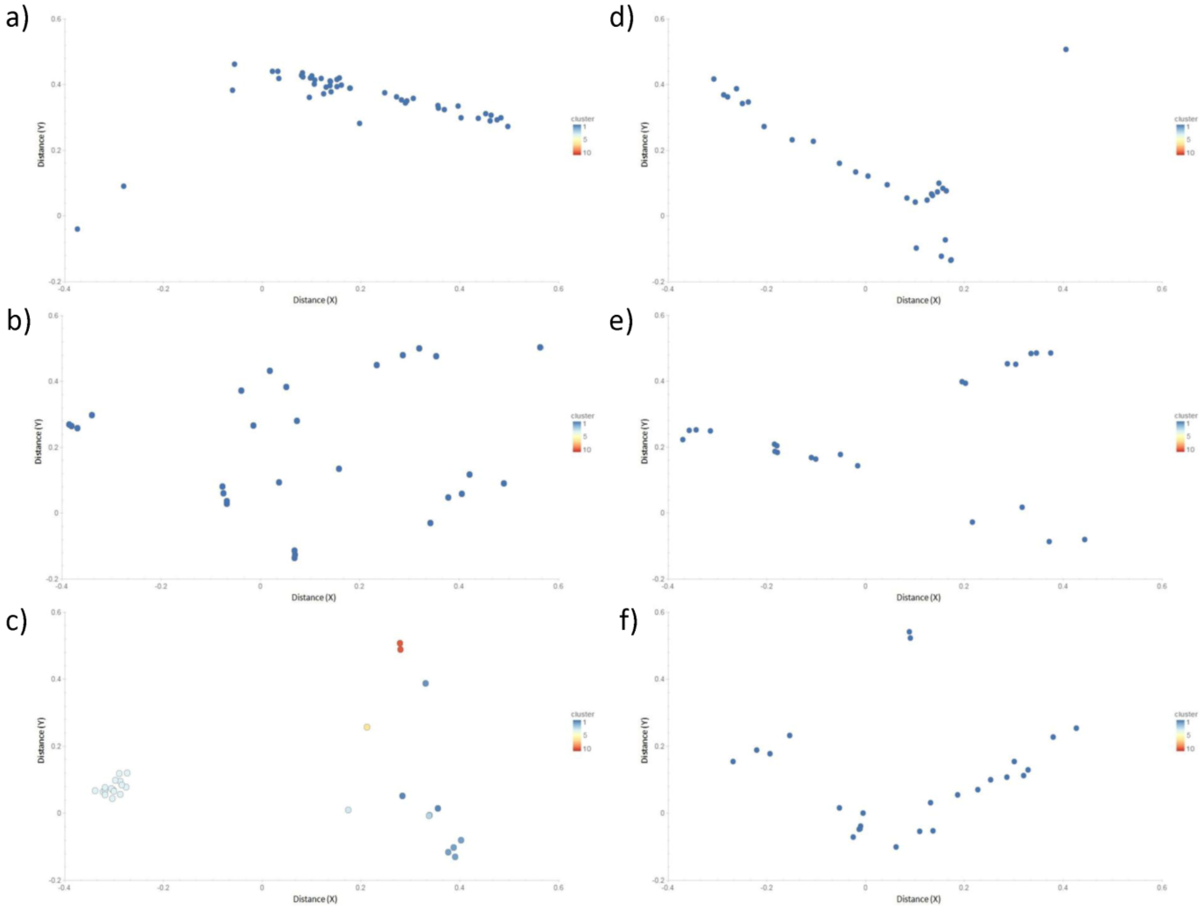

.

Figure S2. Chemical space mapping outline of a) Integrase (IN), b) Protease (PR) and c) Reverse Transcriptase (RT) inhibitors (percentage inhibition) with internal circle showing clustering and 3-D embedding of compounds, middle circle with exact (zoomed) superimposed

cluster and outermost circle with specific MCS of each cluster.

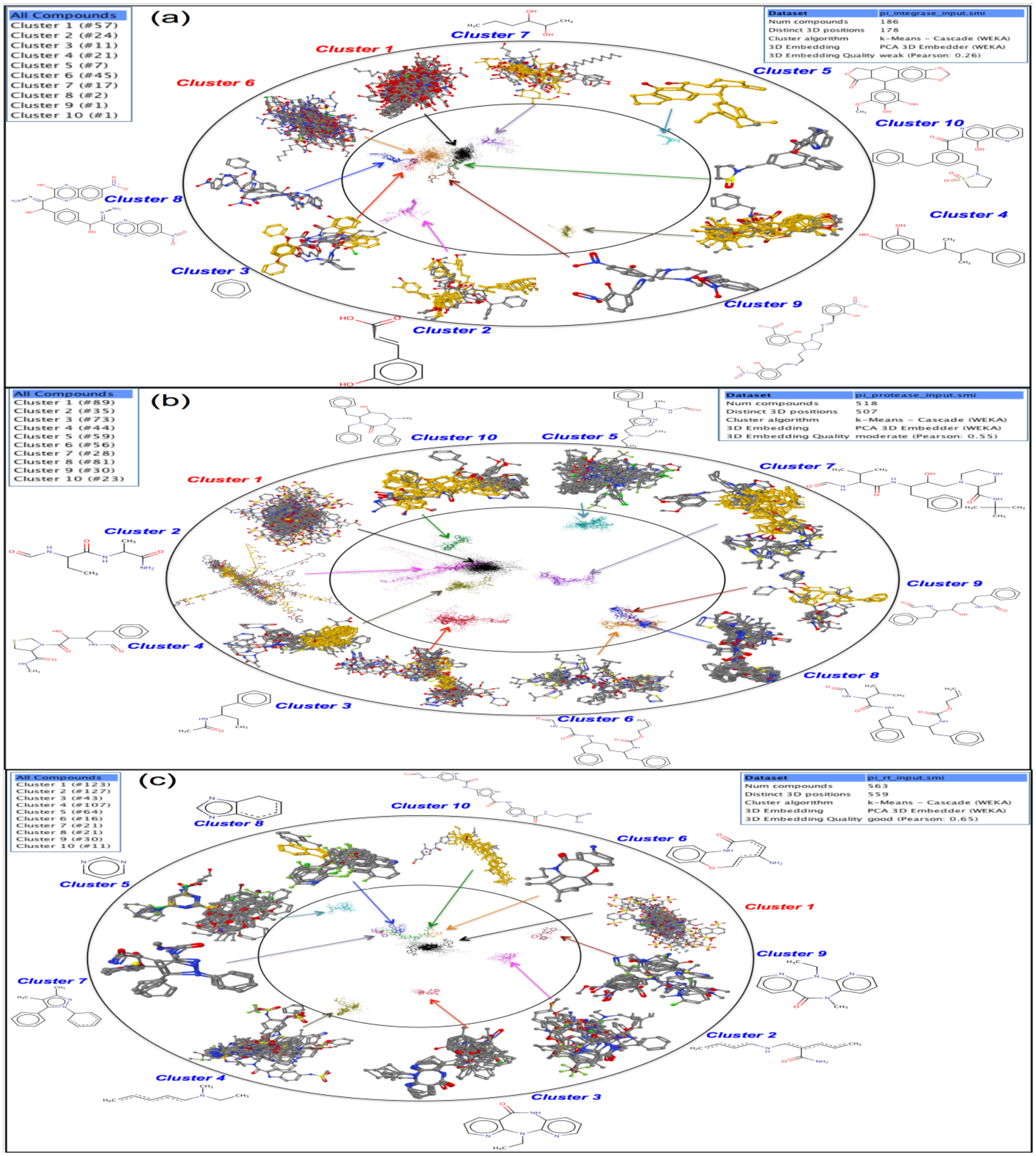

Figure S3. Scatter plot depicting the applicability domain for IC_{50} datasets of a) Integrase (IN), b) Protease (PR) and c) Reverse Transcriptase (RT).

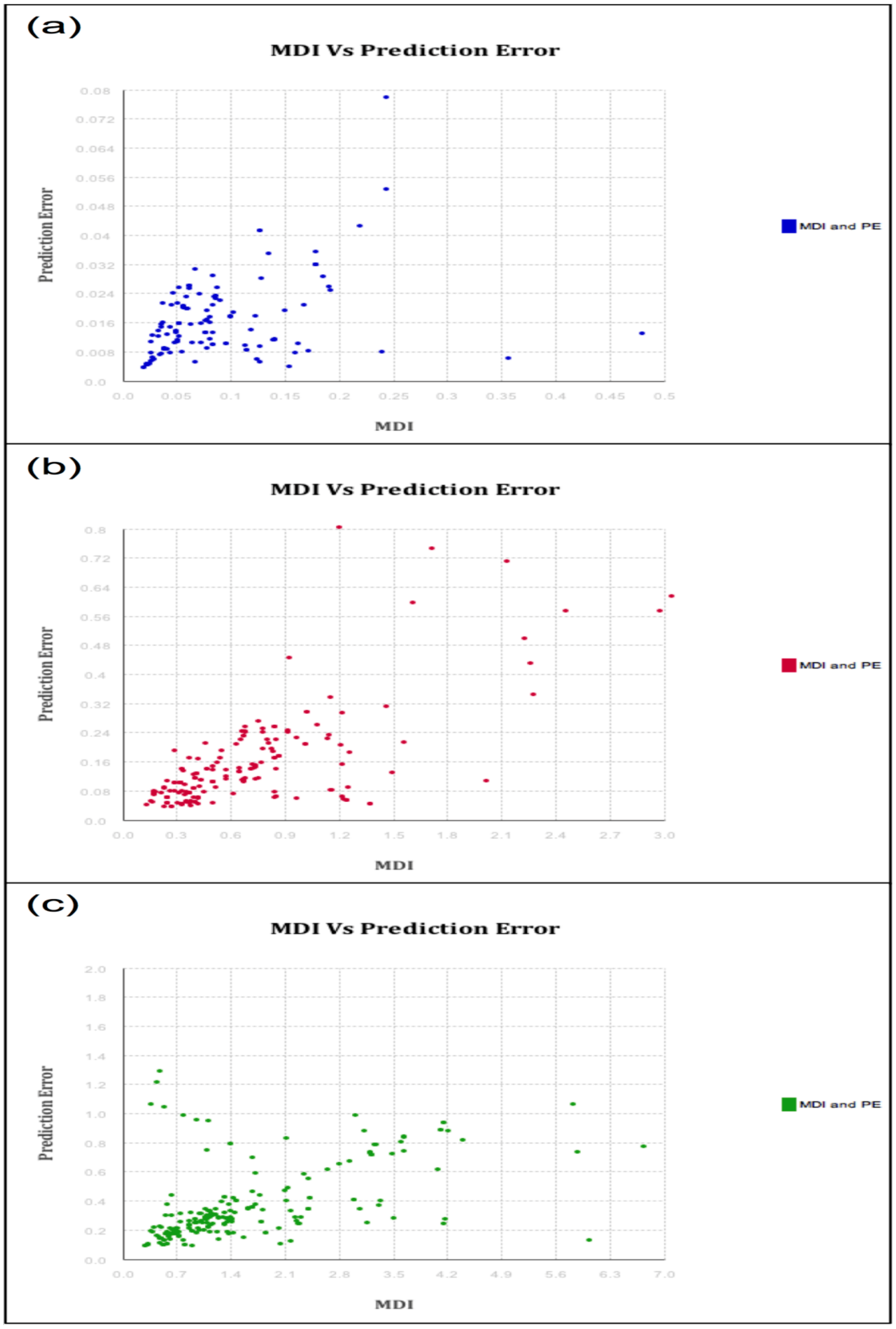

Figure S4. Scatter plot depicting the applicability domain for percentage inhibition datasets of a) Integrase (IN), b) Protease (PR) and c) Reverse Transcriptase (RT).

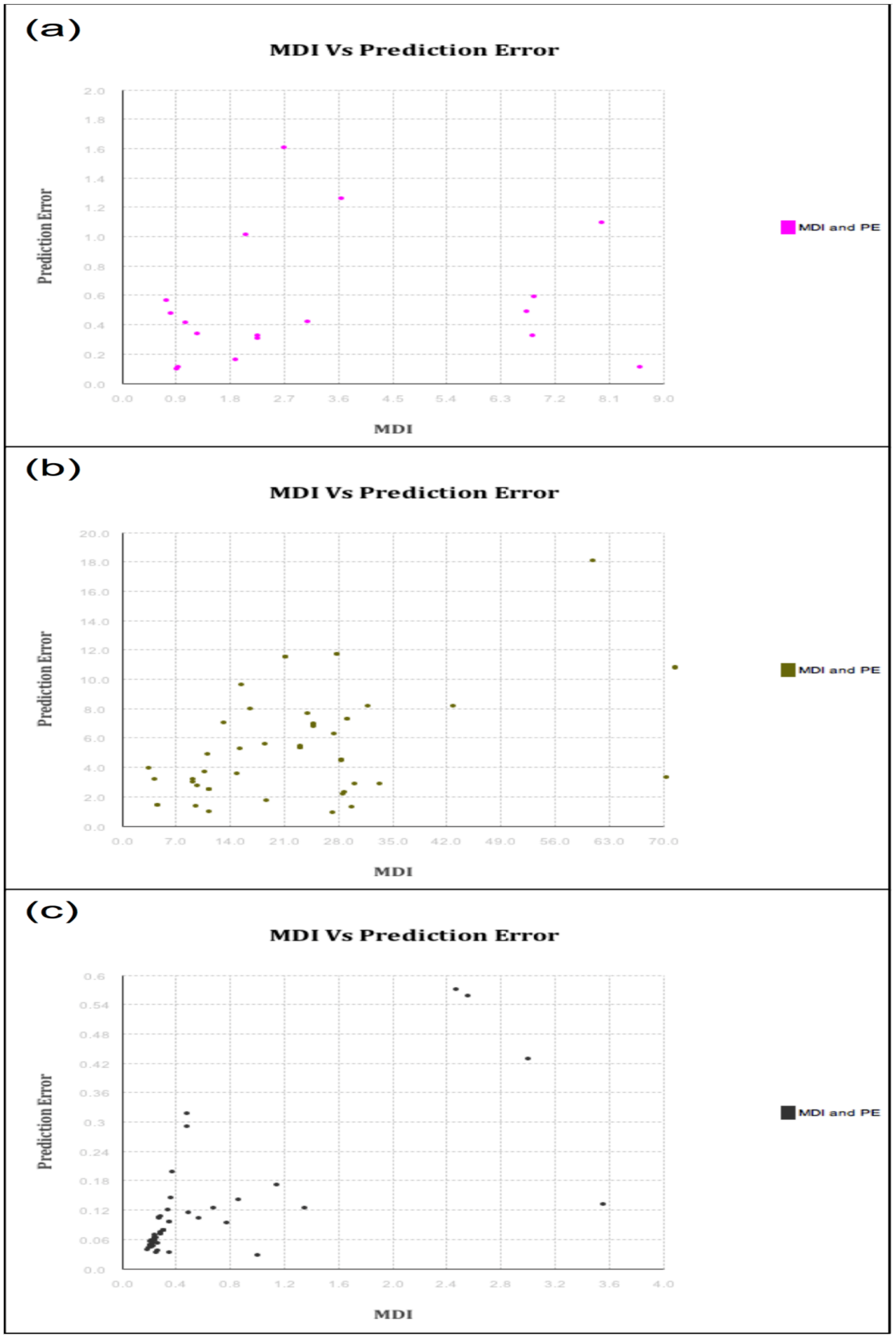
(a)
MDI Vs Prediction Error
Prediction Error
2.0
1.8
1.6
1.4
1.2
1.0
0.8
0.6
0.4
0.2
0.0
0.0 0.9 1.8 2.7 3.6 4.5 5.4 6.3 7.2 8.1 9.0
MDI
MDI and PE
(b)
MDI Vs Prediction Error
Prediction Error
20.0
18.0
16.0
14.0
12.0
10.0
8.0
6.0
4.0
2.0
0.0
0.0 7.0 14.0 21.0 28.0 35.0 42.0 49.0 56.0 63.0 70.0
MDI
MDI and PE
(c)
MDI Vs Prediction Error
Prediction Error
0.6
0.54
0.48
0.42
0.36
0.3
0.24
0.18
0.12
0.06
0.0
0.0 0.4 0.8 1.2 1.6 2.0 2.4 2.8 3.2 3.6 4.0
MDI
MDI and PE
