# Supplementary material for: HIVprotI: an integrated web based platform for prediction and design of HIV proteins inhibitors
Source: J Cheminform. 2018 Mar 9;10:12. doi: 10.1186/s13321-018-0266-y (PMC5845081; doi:10.1186/s13321-018-0266-y)
Supplement: Supplementary file 2 — Additional file 2. Source code of HIVProtI web server. [file 13321_2018_266_MOESM2_ESM.zip › HIVprotI_Source-code/hivprotI/calc_desc_analog.php]

php include("head.php");
$ran=$\_GET['ran'];
$dir="/home/gpsr/webserver/cgidocs/tmp/manojk/hivproti/first\_$ran";
//$x=file\_get\_contents("$dir/input\_min.sdf");
//echo"$x";
echo"<br  
";
echo"

|  |
| --- |
| **Chemical Descriptors of Query Molecules:** |

";
echo"  
  
  
";
shell\_exec("perl filter\_analog.pl $ran");
shell\_exec("perl desc\_filter\_analog.pl $ran");
include("topkey3");
include("$dir/desc\_filter2");
include("bottom");
#shell\_exec("rm $dir/desc\*");
shell\_exec("cp $dir/desc\_filter1 $dir/descriptors\_intermediate.txt");
shell\_exec("cat top\_key\_calc $dir/descriptors\_intermediate.txt > $dir/descriptors.txt");
echo "  
";
#####Predict######
echo"

|  |  |
| --- | --- |
| "; /\*echo " |";\*/
echo "  |

";
###################
?>
php include("foot.php");?
