# Supplementary material for: HIVprotI: an integrated web based platform for prediction and design of HIV proteins inhibitors
Source: J Cheminform. 2018 Mar 9;10:12. doi: 10.1186/s13321-018-0266-y (PMC5845081; doi:10.1186/s13321-018-0266-y)
Supplement: Supplementary file 2 — Additional file 2. Source code of HIVProtI web server. [file 13321_2018_266_MOESM2_ESM.zip › HIVprotI_Source-code/hivprotI/cdw/htc.html]

|  |  |  |
| --- | --- | --- |
| Pubchem-Name: |  | e.g. Aspirin |
| Smiles: |  | e.g. CC(=O)OC1=CC=CC=C1C(=O)O |

The Anatomical Therapeutic Chemical (ATC) classification system, published by the WHO, is used for the classification of drugs.  
  
Our drug classification method is based on 2,650 actual classified drugs (with assigned ATC codes) by the WHO.  
  
The method considers 2D, fragment and 3D similarity to classify your input compound.

  

**No compound found. Please try again.  
PubChem notation required!**

|  |
| --- |
|  |

|  |

  
