# Supplementary material for: HIVprotI: an integrated web based platform for prediction and design of HIV proteins inhibitors
Source: J Cheminform. 2018 Mar 9;10:12. doi: 10.1186/s13321-018-0266-y (PMC5845081; doi:10.1186/s13321-018-0266-y)
Supplement: Supplementary file 2 — Additional file 2. Source code of HIVProtI web server. [file 13321_2018_266_MOESM2_ESM.zip › HIVprotI_Source-code/hivprotI/cdw/samples/index.html]

Periodic Table
  
Ball and Stick Rotator
  
2D Sketcher (Full Sketcher)
  
2D Sketcher (Single Molecule Sketcher)
  
Mass Spectrum
  
Morphine Wire Transformer
  
Caffeine Viewer
  
WebGL Transformer 1CRN
  
WebGL Transformer 2OEY
  
WebGL Transformer DDT
  
WebGL Transformer MAZ
